# Neonatal hypoxic-ischemic encephalopathy after acute carbon monoxide intoxication during pregnancy. A case report and brief review of the literature

**DOI:** 10.3389/fped.2023.1264855

**Published:** 2023-11-03

**Authors:** Cristina Tuoni, Giulia Nuzzi, Rosa Teresa Scaramuzzo, Simona Fiori, Luca Filippi

**Affiliations:** ^1^ Neonatology Unit, Azienda Ospedaliero-Universitaria Pisana, Pisa, Italy; ^2^ Department of Clinical and Experimental Medicine, Section of Pediatrics, University of Pisa, Pisa, Italy; ^3^ Department of Clinical and Experimental Medicine, University of Pisa, Pisa, Italy; ^4^ Department of Developmental Neuroscience, IRCCS Stella Maris Foundation, Pisa, Italy; ^5^ Division of Neonatology and NICU, Department of Clinical and Experimental Medicine, University of Pisa, Pisa, Italy

**Keywords:** carbon monoxide intoxication, hypoxic-ischemic encephalopathy, fetal brain damage, carboxyhemoglobin, cerebral palsy

## Abstract

Carbon monoxide (CO) poisoning during pregnancy is a rare occurrence, associated with high maternal and fetal mortality rates. As CO can cross the placenta, leading to intrauterine hypoxia, CO intoxication can result in neurological sequelae and neurologic complications in fetuses who survive. We report a case of a preterm newborn acutely exposed to CO in-utero and delivered by emergent cesarean section at the 31st week of gestation due to the severe burns suffered by the mother following an indoor boiler explosion. As CO has serious adverse effects both on the mother and fetus, it is important to recognize and treat poisoning in a timely manner. Despite maternal blood CO levels, CO intoxication at critical stage of central nervous system development can lead to hypoxic-ischemic lesions, thus interdisciplinary care and follow up for these patients are mandatory.

## 1. Introduction

Carbon monoxide (CO) is an odorless, tasteless, colorless, nonirritating gas formed by hydrocarbon combustion. CO poisoning is estimated to occur in 50,000 people annually in the United States with approximately 1,000–1,300 deaths from CO poisoning annually (1). Acute perinatal CO poisoning is a rarely reported occurrence, which may however represent a cause of neonatal hypoxic-ischemic encephalopathy. Cases of CO poisoning in pregnancy are particularly relevant because the fetus may be more affected than the mother by CO exposure, due to the higher affinity of fetal hemoglobin for CO. Indeed, the affinity of hemoglobin for CO is 200 times its affinity for oxygen, and this affinity increases for fetal hemoglobin. Thus, in a setting of CO poisoning, fetal carboxyhemoglobin (COHb) level will be higher than that of the mother, and clearance of CO will be up to 5 times slower (2). Oxygen is displaced from hemoglobin, leading to tissue hypoxia by inhibiting transport, delivery and utilization of oxygen (3). Herein, we present a case of a preterm newborn, delivered at 31 weeks of gestational age by emergent cesarean section following acute CO intoxication by the mother. Given the in-utero exposure to CO, the patient suffered from moderate hypoxic-ischemic encephalopathy. The literature on its pathophysiology, diagnosis, and management is also reviewed.

## 2. Case description

**Figure 1:**
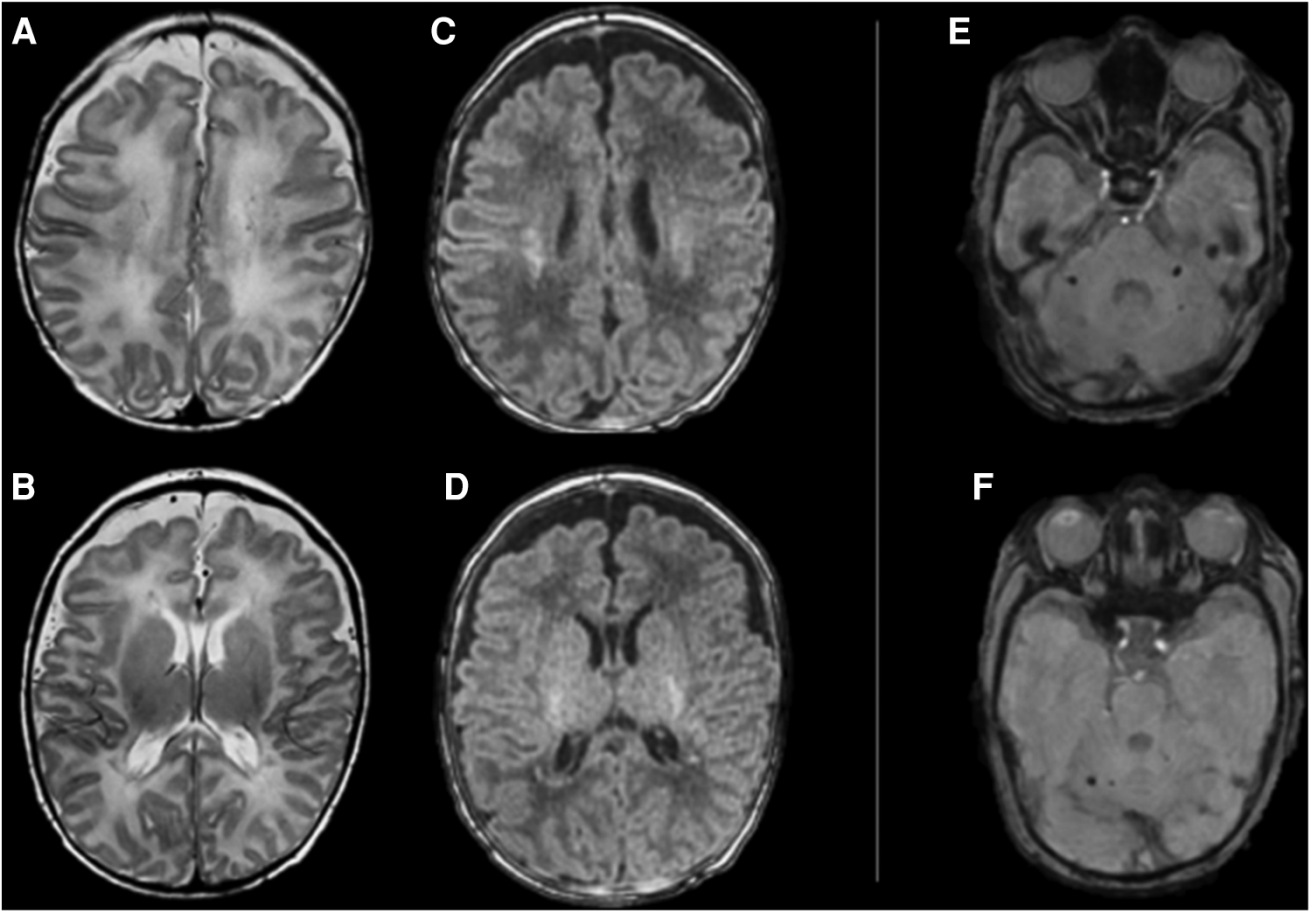
Brain MRI performed at 42 weeks of gestational age shows supratentorial (**A–D**) and infratentorial (**E,F**) pattern of brain injury. From left to right, supratentorial axial FSE T2 images (**A–D**) showing hypointense punctate confluent white matter lesions in the centrum semiovale (**A**) and in the periventricular white matter (**B**); axial MP-RAGE T1 images showing hyperintense signal corresponding to the abovementioned T2 signal abnormalities (**C,D**). Infratentorial axial Susceptibility Weighted images (**E,F**) shows rounded small spots of signal intensity in the cerebellar peduncles bilaterally (**E**) and in the right dentate region (**F**).

A 26-year-old no smoker pregnant woman (gravida 1, para 0), with no significant medical history, was admitted to the Emergency Department (E.D.) at 31 weeks gestational age approximately 1 h after being exposed to an indoor boiler explosion. Upon arrival at the E.D., she had been ventilated in the previous hour with 100% oxygen and her carboxyhemoglobin (CO-Hb) was 5.3%. Emergent cesarean section was performed due to findings of fetal distress and 2nd degree burns in 80% of the mother's body. The woman's vital parameters showed a blood pressure of 140/110 mmHg, with a heart rate of 105 bpm and oxygen saturation of 96%. At birth the newborn's heart rate was below 60 bpm, and he was hypotonic, with absence of crying and spontaneous breathing, with a bright red discoloration of the skin. After aspiration of the airways, mask ventilation was performed for approximately 30 s with 100% oxygen in suspicion of acute carbon monoxide intoxication. Given the persistent absence of spontaneous breathing with heart rate below 100 bpm, endotracheal intubation was carried out and cardiopulmonary resuscitation was performed for the establishment of adequate heart rate and respiration, obtained after the administration of intravenous epinephrine. Apgar scores at 1 min, 5 min and 10 min were 3, 4 and 6, respectively. The infant's arterial cord gas result was the following: pH = 7.29, PCO2 = 47 mmHg, pO2 = 25 mmHg, bicarbonate = 22.6 mmol/L Lactate = 2.6 mmol/L, and carboxyhemoglobin = 4.0%. The newborn was transferred to the Neonatal Intensive Care Unit (NICU) on mechanical ventilation with 100% oxygen. In the next days serial blood gas analyses showed a gradual and progressive reduction of CO-Hb values (Table 1), until a complete normalization was reached in the 8th day of life, when the baby was extubated, and non-invasive respiratory support was continued for 10 days. His laboratory data were unremarkable, and his hospital stay was uneventful. Serial cranial ultrasound showed bilateral persistent non-homogeneous periventricular hyperechogenicity. Brain magnetic resonance imaging was performed and revealed a supratentorial pattern of confluent punctate white matter lesions in the deep and periventricular white matter associated with infratentorial small foci of hemorrhagic injury in the cerebellum (Figure 1). At 3 months of life on neurological objectivity, the spontaneous movement pattern was characterized by sporadic fidgety movements with monotonous repertoire; mild four-limb hypertension was appreciated, with increased deep tendon reflexes.

**Table 1:** Serial blood gas analyses data of the newborn during the hospital stay in NICU.

	FiO_2_	pH	pCO_2_	pO_2_	HCO_3_^−^	BE	Lac	COHb	SO_2_	Hb
27/10 h 18	45	7.40	38	41	23.5	−1.0	3.0	3.9	91.2	16
27/10 h 21	35	7.24	55	20	23.6	−4.7	2.8	2.7	45.5	15.7
28/10 h 6	35	7.39	32	35	19.4	−4.4	4.4	2.6	87.2	16.4
28/10 h 12	30	7.33	36	31	19	−6.2	4.9	2.5	82.7	16.3
28/10 h 23	26	7.36	37	45	20.9	−4.0	4.2	2.3	88.6	14.3
29/10 h 8	24	7.35	37	45	20.4	−4.7	4.8	2.2	79.6	11.9
30/10 h 8	24	7.38	35	54	20.7	−3.8	3.1	2.1	91.2	13
31/10 h 8	24	7.32	32	48	16.5	−8.4	4.3	2.4	88.5	15.2
01/11 h 8	24	7.25	42	42	18.4	−8.5	2.3	2.4	84.2	15
02/11 h 8	21	7.26	44	49	19.7	−7.3	1.4	2.0	87.9	15
03/11 h 8	21	7.29	42	54	20.2	−6.2	1.4	2.1	91.7	14.5
06/11 h 8	21	7.33	49	51	25.8	−0.7	1.5	1.8	90	13.2
09/11 h 8	21	7.40	48	43	29.7	4.1	1.2	1.7	83.6	11.3
15/11 (art)	21	7.44	44	75	29.9	5.1	1.6	1.1	96.3	10.3

## 3. Discussion

Although CO poisoning in the adult population is well described, little information exists in infants and pregnant women. CO poisoning is indeed a common cause of morbidity and mortality but is relatively rare during pregnancy. The frequency of acute CO poisoning in pregnancy is difficult to estimate, but is associated with a maternal mortality rate of between 19% and 24% and a fetal mortality rate of between 36% and 67%, with variability arising from the severity of maternal poisoning and gestational age (4). CO spreads rapidly in the blood through the lungs leading to hypoxemia through the formation of carboxyhemoglobin. Indeed, given the higher affinity of hemoglobin for CO than for oxygen, at the molecular level there is competition between CO and oxygen for hemoglobin (5).

Binding of CO by hemoglobin leads to a left shift of the hemoglobin dissociation curve and alters the shape of the curve toward a more hyperbolic form, decreasing the release of oxygen to the tissues. In addition, CO binds to heme proteins such as cytochrome c oxidase, impairing mitochondrial function and thus contributing to hypoxia (6). CO also causes inflammation by increasing levels of cytosolic heme and the protein heme oxygenase-1, resulting in intracellular oxidative stress, causes platelet-neutrophil aggregation and neutrophil degranulation, with the production and release of reactive oxygen species (ROS) that cause oxidative stress, lipid peroxidation and cellular apoptosis (7, 8). Acute CO toxicity in pregnancy causes hypoxia of fetal tissue through two mechanisms, i.e., transplacental passage of CO and reduced levels of oxygen release from maternal hemoglobin. CO diffuses across the placenta either by passive diffusion or through a transporter-mediated mechanism and this diffusion increases with gestational age and in proportion to fetal weight, as a result of changes in placental blood flow velocity and maternal hemoglobin concentration. Because of the lower baseline PaO2 of fetal blood compared with maternal blood, and the natural left shift of the fetal hemoglobin dissociation curve, the fetus is more susceptible to CO-related insults (9). As maternal CO-Hb increases and blood oxygen content decreases, both hemoglobin's ability to release oxygen and oxygen transport across the placenta are drastically reduced. Therefore, oxygen carried through the umbilical vein decreases, leading to fetal hypoxia. In addition, the dissociation of maternal CO-Hb creates a pressure gradient between maternal and fetal blood, causing placental CO diffusion. Once the CO reaches the fetal blood, it easily combines with fetal hemoglobin. As a result, oxygen is displaced from hemoglobin and oxygen-carrying capacity decreases leading to cellular hypoxia. Therefore, inhalation of CO by the mother results in intrauterine hypoxia by inhibiting transport, delivery, and utilization of oxygen. Binding ability of CO to fetal hemoglobin is almost 3 times greater than the adult hemoglobin, thus fetal CO-Hb levels are about 10%–15% higher than maternal levels (10).

As already cited, the mechanisms of CO-mediated toxicity are complex, because CO not only displaces oxygen from hemoglobin, inhibiting oxygen-carrying capacity of hemoglobin, but also acts as an asphyxiant at a cellular level by stimulating lipid peroxidation, oxygen-free radical production, and reperfusion injury (11). Fetal outcome is difficult to estimate, but is thought to be proportional to the severity of maternal symptoms and the time of gestation, while appears to not be correlated to the mother's level of CO-Hb (10). Indeed, maternal CO-Hb levels do not accurately reflect fetal hemoglobin or tissue levels, thus they have little correlation with the newborn outcome (11, 12). Fetus is particularly susceptible to the effects of CO, and during the late gestational stage the fetal brain seems to be more sensitive to CO toxicity (10, 13). During the late stage, congenital abnormalities are rare, but death or severe neurological sequelae may occur (14). Studies found that the most vulnerable brain areas for CO injury are those containing white matter (especially periventricular) and the brainstem, followed by the thalamus and cerebral cortex (15, 16). As shown in the reported case, our patient was exposed to CO at the 31st week of gestation and, as expected, hypoxic-ischemic brain lesions appeared instead of microcephaly or central nervous system (CNS) development disorders. The only possible non-teratogenic treatment for pregnant women with CO poisoning is hyperbaric oxygen therapy (17), as shown by Elkharrat et al. (18) Hyperbaric oxygen therapy (HBO), i.e., the therapeutic use of 100% pure oxygen at above-atmospheric pressure, is the standard treatment for acute CO poisoning, as oxygen accelerates the dissociation of CO from hemoglobin, improving tissue oxygenation. For this reason, it has been considered in recent years to prevent the cognitive sequelae of CO poisoning, even in newborns (19). Anyway, HBO therapy leads to side effects, such as CNS oxygen toxicity leading to seizures and retrolental fibroplasia in preterm neonates delivered before 35 weeks of gestation, that should be considered before starting this treatment. Decisions regarding medical management of a CO intoxicated pregnant woman and her fetus can be difficult. Given the rarity of this condition, no specific treatment guidelines are available from the American College of Obstetricians and Gynecologists (ACOG) or American Academy of Pediatrics (19). Options for treatment include normobaric oxygen treatment, hyperbaric oxygen treatment, delivery and subsequent treatment of the neonate with 100% oxygen. If HBO is not available, a CO intoxicated pregnant woman should be administered 100% oxygen and monitored for the fetus wellbeing closely. In our case, at the time of acute CO intoxication, the mother was at the 31st week of gestation which had a very high chance of survival with more benefits than risks in performing an emergent caesarean section immediately; therefore, after the stabilization of the mother, delivery option should be thought as soon as possible.

## 4. Conclusion

We conclude that CO intoxication at critical periods of human brain development can lead to brain injury and that signs of encephalopathy may develop in the infant during long-term follow-up. Therefore, all infants with a history of exposure to CO *in utero* should be evaluated with serial cranial imaging, even if neurologic examinations at birth and in the first days of life seems normal. We also suggest making aware the family of possible neurodevelopmental sequelae in the following months, thus interdisciplinary care for these patients is mandatory.

## Data Availability

The original contributions presented in the study are included in the article/Supplementary Material, further inquiries can be directed to the corresponding author.
